# Supplementary material for: Mapping CRISPR spaceromes reveals vast host-specific viromes of prokaryotes
Source: Commun Biol. 2020 Jun 22;3:321. doi: 10.1038/s42003-020-1014-1 (PMC7308287; doi:10.1038/s42003-020-1014-1)

Supplementary figure 1

1000-fold bootstrap resampling of the set of spacers from figure 2b (all completely sequenced genomes) and figure 2c (154 genomes with spacers matches in viruses). Lines represent original values: blue, real spacers matching viral genomes; dark blue, mock spacers matching viral genomes; red, real spacers matching the host genome; dark red, mock spacers matching the host genome. Box plots represent results of bootstrapping of these values.

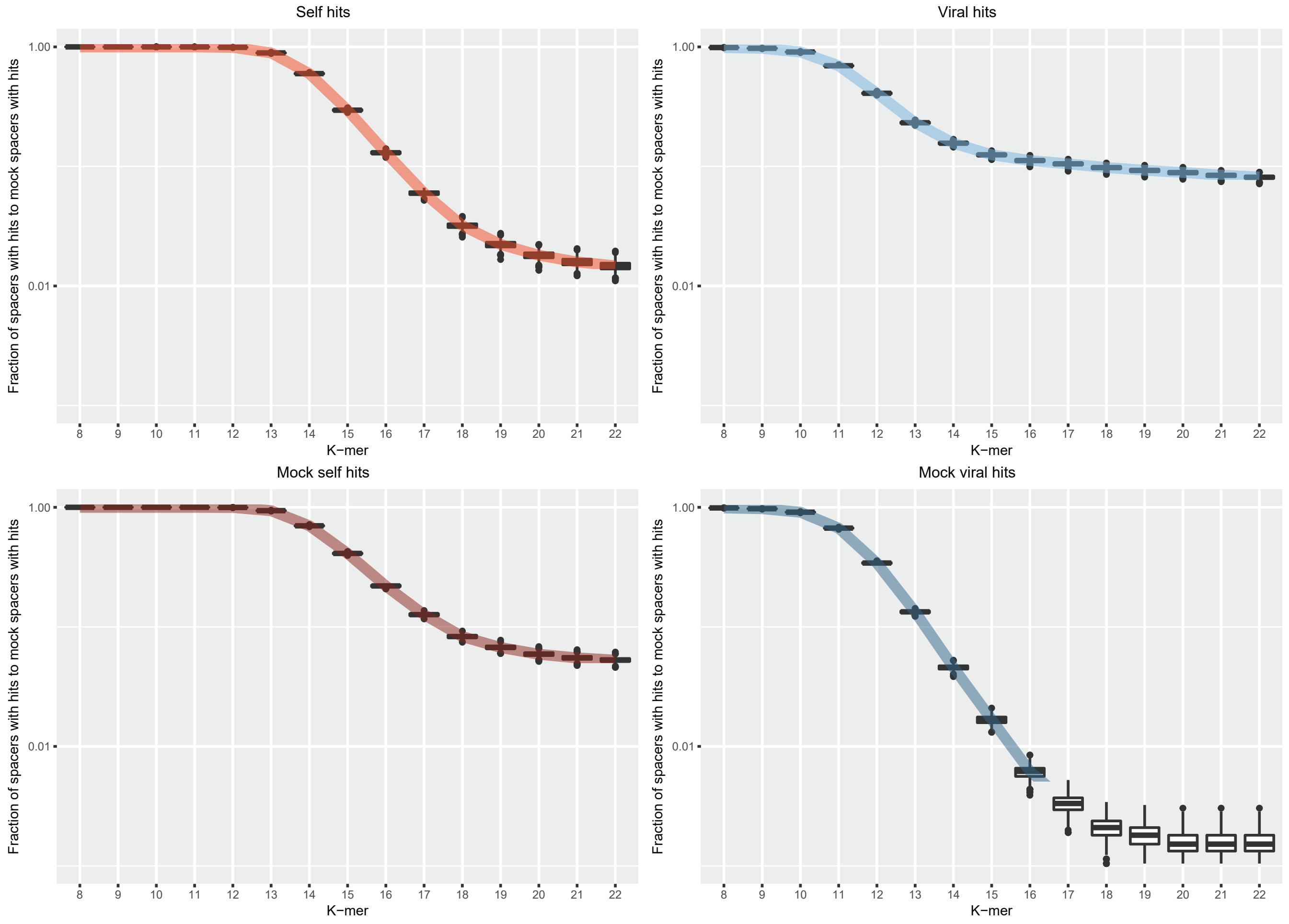

Bootstrap for values shown on figure 2b (all completely sequenced genomes)

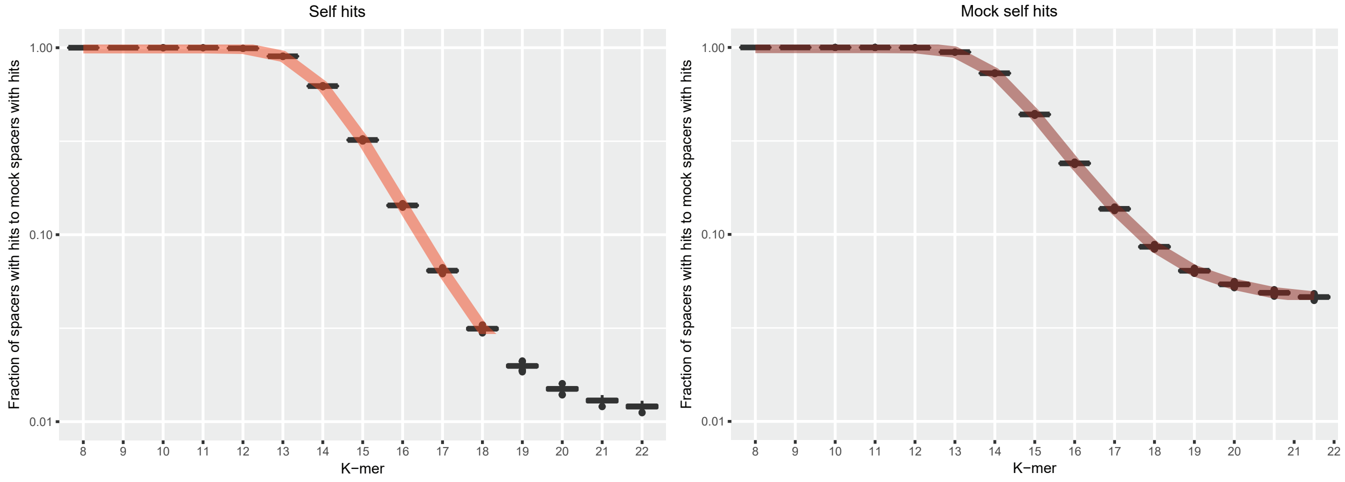

Supplementary figure 2
Fractions of virus-matching and self-matching spacers (of the total number of spacers) for the scrambled spacers set for 154 genomes containing CRISPRs with identifiable protospacers in viruses. Line colors: blue, real spacers matching viral genomes; dark blue, mock spacers matching viral genomes; red, real spacers matching the host genome; dark red, mock spacers matching the host genome.

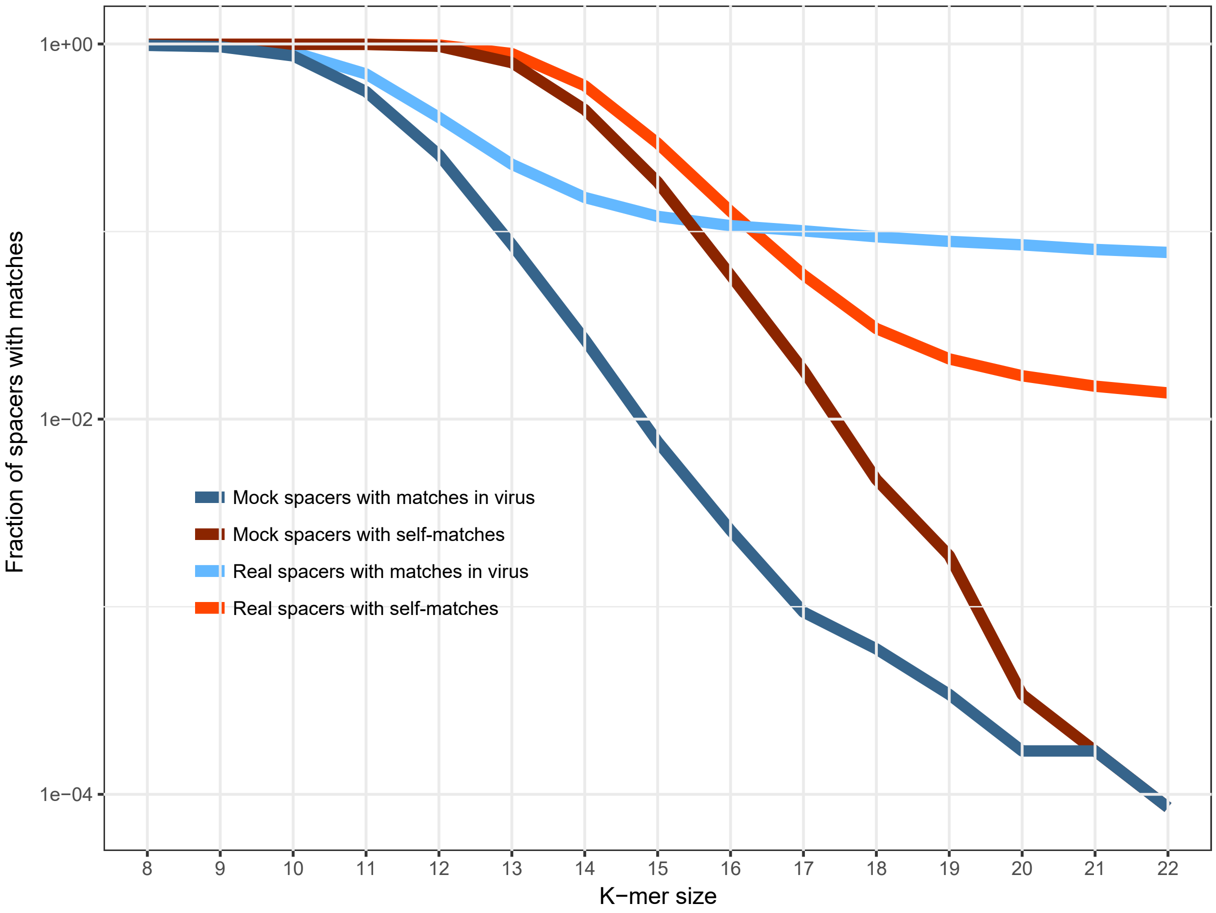

Supplementary figure 3
1000-fold bootstrap resampling of the set of spacers from figure 3 (genomes with well sampled viromes).

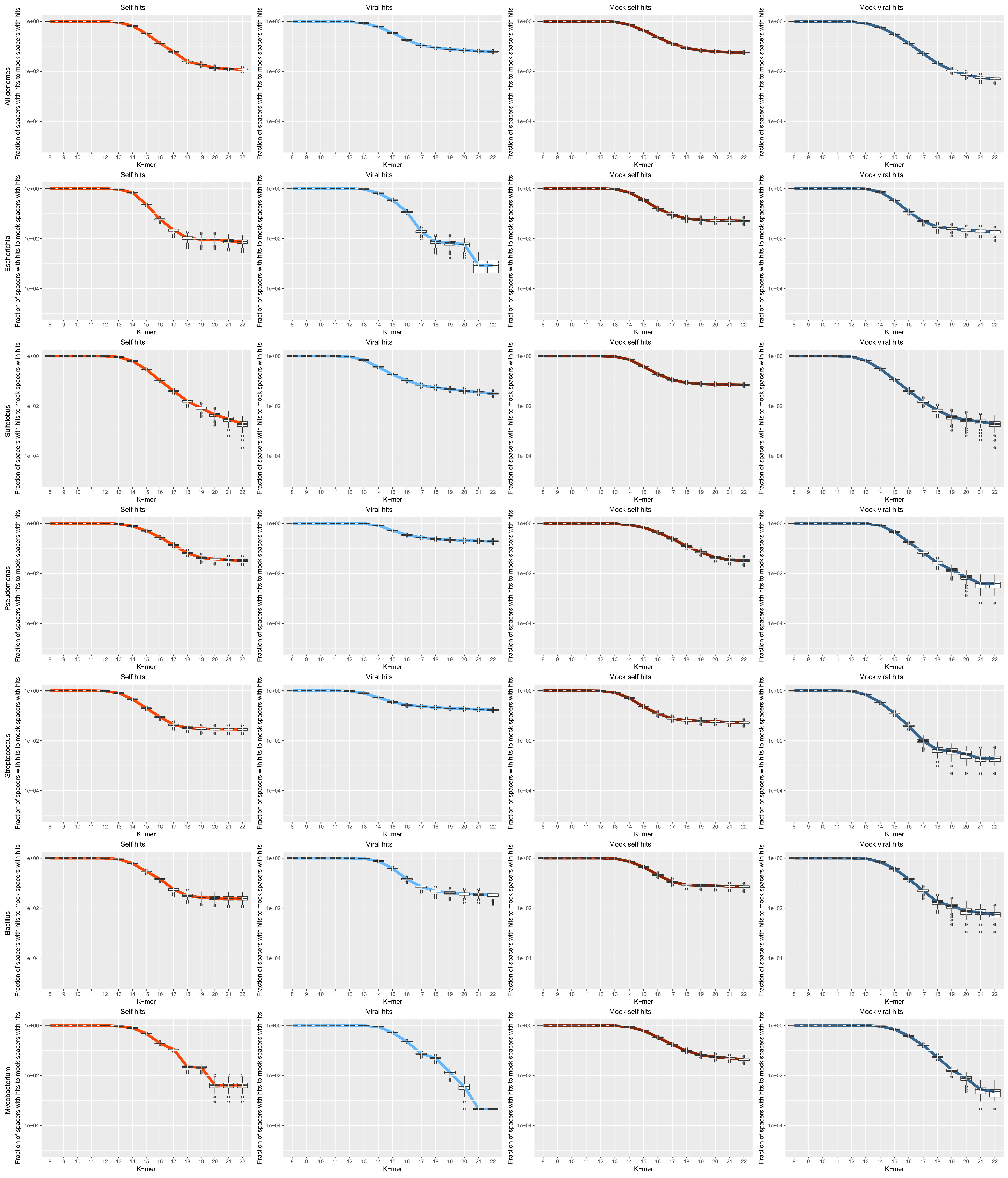

Supplementary figure 4

1000-fold bootstrap resampling of the set of spacers from figure 4 (data from adaptation experiments).

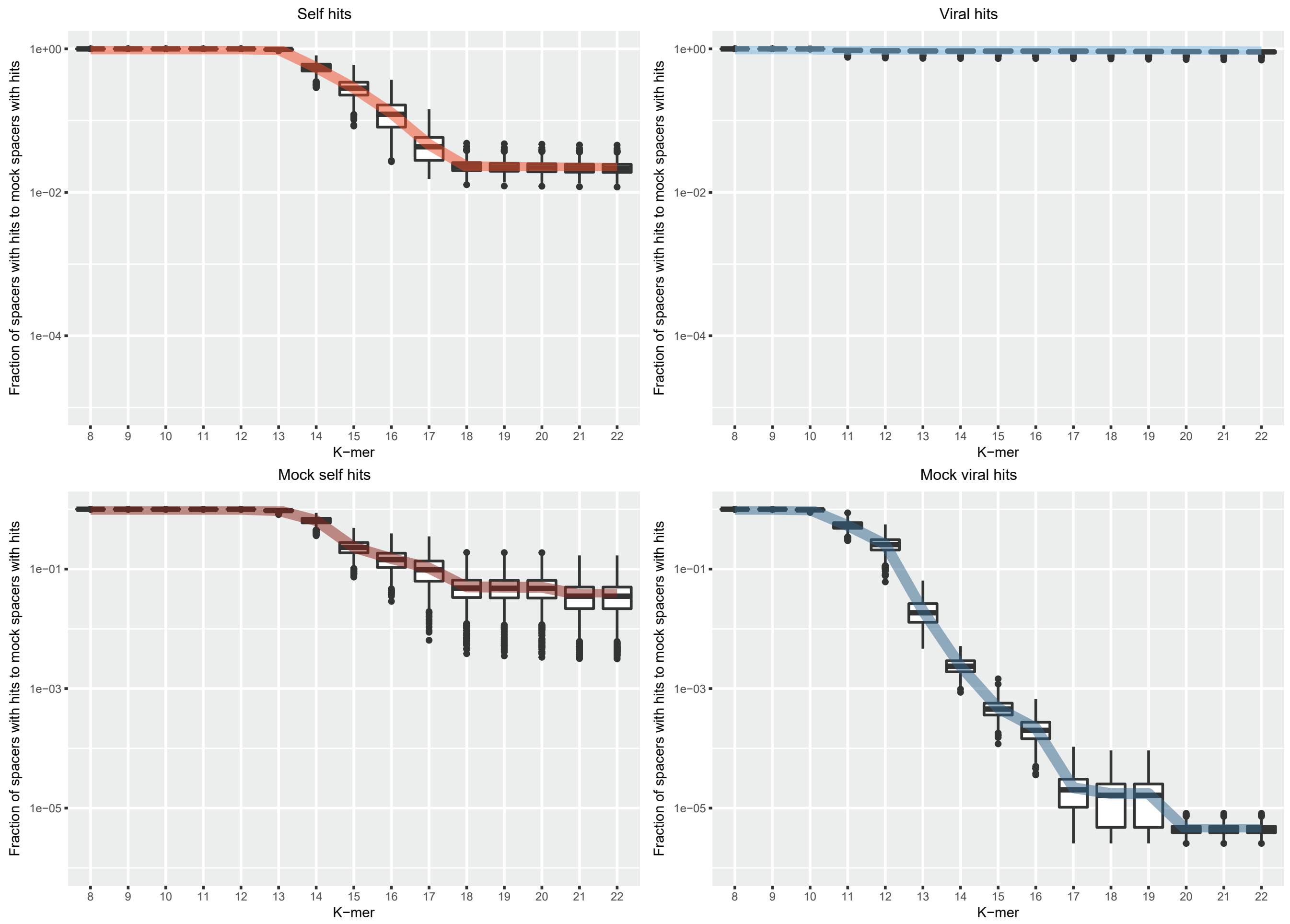

Bootstrap for values shown on figure 4 for lambda phage

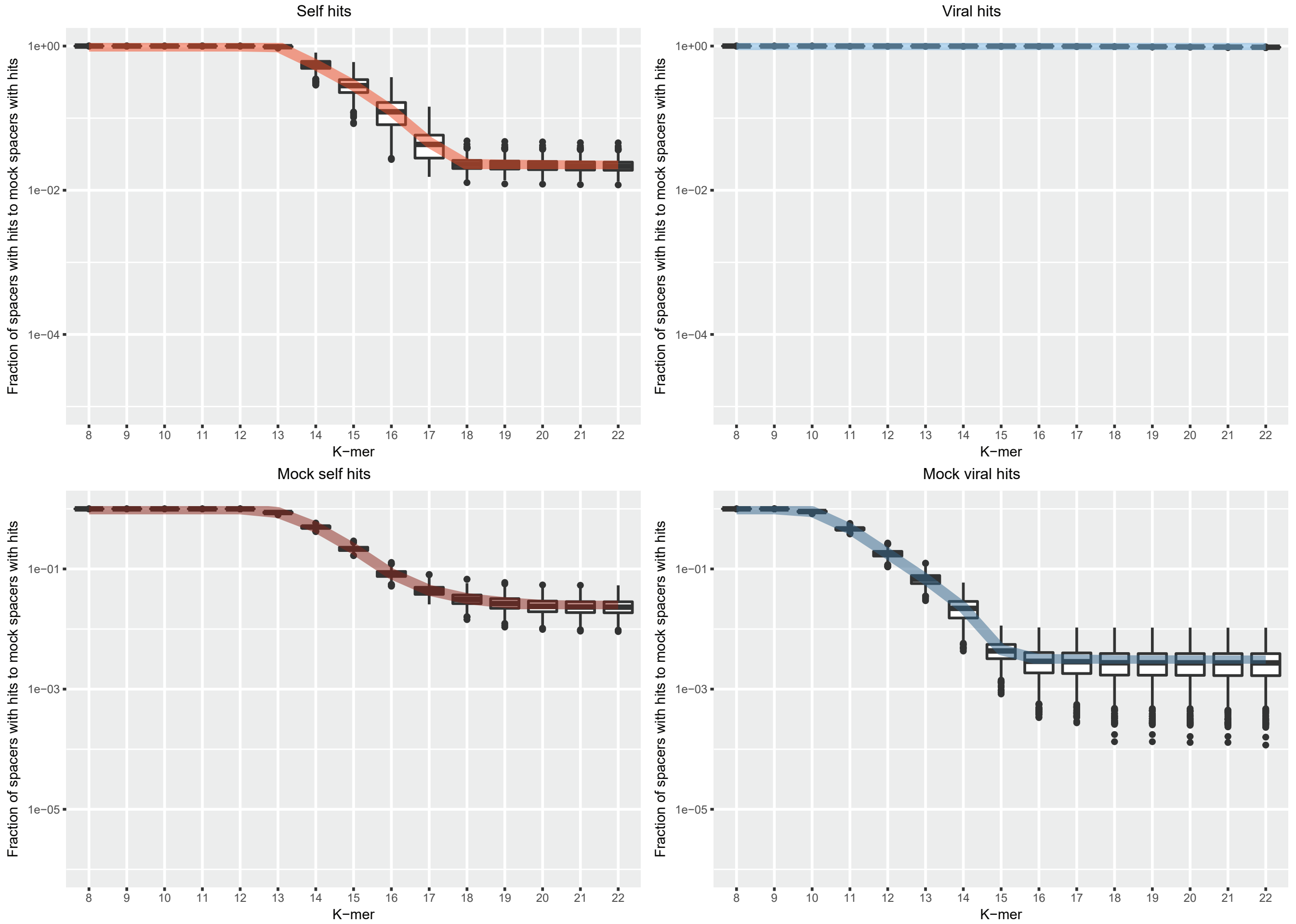

Supplement: Supplementary file 1 — Supplementary Information [file 42003_2020_1014_MOESM1_ESM.pdf]
