# Supplementary material for: Mapping CRISPR spaceromes reveals vast host-specific viromes of prokaryotes
Source: Commun Biol. 2020 Jun 22;3:321. doi: 10.1038/s42003-020-1014-1 (PMC7308287; doi:10.1038/s42003-020-1014-1)
Supplement: Supplementary file 2 — Description of Additional Supplementary Files [file 42003_2020_1014_MOESM2_ESM.pdf]

Description of Additional Supplementary Files

File Name: Supplementary Data 1
Description: Coordinates for matches and possible annotation for all mock spacers with >= 5 matches in self genome.

File Name: Supplementary Data 2
Description: Characteristics of self-matching spacers in genomes containing >= 5 self-matches.

File Name: Supplementary Data 3
Description: Coordinates for matches and possible annotation for all 22-mers from mock spacers with matches in viral genomes

File Name: Supplementary Data 4
Description: Numbers of different PAMs for protospacers found in in adaptation experiments for self and viral matches.
